# Supplementary material for: The Clinical and Psychosocial Outcomes for Women Who Received Unexpected Clinically Actionable Germline Information Identified through Research: An Exploratory Sequential Mixed-Methods Comparative Study
Source: J Pers Med. 2022 Jul 7;12(7):1112. doi: 10.3390/jpm12071112 (PMC9315752; doi:10.3390/jpm12071112)
Supplement: Supplementary file 1 [file jpm-12-01112-s001.zip › jpm-1736427-supplementary.pdf]

Attachment D – Group 1 **life**pool psychosocial survey
[Note – this survey will be mounted onto REDCap, the formatting here is a guide only]

Information and consent to participate

You have been invited to participate in a study being conducted at the Peter MacCallum Cancer Centre: *'The psychosocial impact of receiving genetic findings from research: experiences of* ***life****pool participants*.'

This project aims to explore the experiences of women who have received genetic findings from the **life**pool project. We are specifically interested in how you consider genetic testing, risk management, risk perception, adaptation and family communication.

You have been invited to participate in this study because you have been identified as an individual who has received genetic findings from **life**pool. Your opinions, views and experiences are valuable to us, and we greatly appreciate your participation.

Please read about the study in the information below. By clicking "Yes" at the bottom of this page you are providing consent to participate in this research study.

This study has been approved by the Peter Mac Human Research Ethics Committee.

What will I be asked to do?

Should you agree to participate, you will be asked to complete a **survey** and an **interview**. The **survey** has 6 sections and will take about 20-30 minutes to complete. By clicking "Yes" at the bottom of this page you can access the survey and you consent to participating in this research. You can complete the survey over more than one session if needed. If so, you will need to make note of your individual Return Code to ensure your answers are saved and you continue where you left off.

The **interview** is expected to take 30-60 mins and can be conducted over the phone or in-person. If in person, you may select the time and venue. With your permission, the interview will be audio recorded. The recording of the interview will be transcribed by a professional transcription service and analysed to develop the results. By clicking "Yes" at the bottom of this page you consent to us contacting you to organise an interview.

There are no costs associated with participating in this research project, nor will you be paid. At the end of the survey you can choose to download a PDF of your completed survey. You may also have a copy of your interview transcript.

How will my confidentiality be protected?

All identifying information about you will be removed so that it is de-identified. Your responses to the survey and/or interview will be stored on a password-protected computer at the Peter MacCallum Cancer Centre in Melbourne, Australia for a minimum period of 5 years. At the end of this storage period all the data will be disposed of by deleting all computer files and backup files.

What are the benefits of participating?

By participating in this research study, you will be contributing to the support of individuals who receive genetic findings from research. There are no expected personal benefits to you the participant.

What are the risks of participating?

In the course of answering questions in the survey or the interview you may experience feelings of anxiety or discomfort. If you do experience any of these feelings, you may contact one of the research team members below, who can organise any counselling or support.

Do I get a copy of the study findings?

At the end of the study we will publish relevant findings in peer-reviewed journals and inform you of the outcome of the study via email.

Where can I get further information?

Should you require any further information, or have any concerns about the content of this survey, please do not hesitate to contact:

Research Assistant:	Lyon Mascarenhas
Telephone:	(03) 8559 6189
Email:	Lyon.Mascarenhas@petermac.org
Available:	Monday, Tuesday and Friday (9am – 5pm)
Principal Investigator:	Dr Laura Forrest
Telephone:	(03) 8559 6191
Email:	Laura.Forrest@petermac.org
Available:	Tues, Wed & Thurs (9am – 5pm)

Should you have any concerns about the conduct of the project, you are welcome to contact the Executive Officer, Human Research Ethics, Peter MacCallum Cancer Centre (P: 03 8559 7540 | E: ethics@petermac.org).

How do I agree to participate?

By selecting the **YES** checkbox below, you agree to consent to participating in this study.

Consent to participate

Do you consent to participate?

- ☐ Yes
- ☐ No

About you

1. Please enter your date of birth (DD/MM/YYYY): ______________________

2. Please enter your postcode: ______________________

3. Please select your highest attained level of education:

☐ Year 11 or below
☐ Year 12
☐ Other Qualifications (non-school)
☐ Certificate level
☐ Advanced Diploma or Diploma
☐ Bachelor Degrees and above*
*includes Graduate Diplomas or Certificates and Postgraduate Degrees

4. What is your current household income?

☐ 0-$18,000
☐ $18,001 - $37,000
☐ $37,001 - $90,000
☐ $90,001 - $180,000
☐ $180,001 and over

5. What is your relationship status?

☐ Single
☐ Married or De facto
☐ Widowed
☐ Separated or Divorced
☐ Other:_____________

6. Do you have any children?

☐ Yes (Go to Q7-8)
☐ No

7. How many daughters? ______________________

8. How many sons? ______________________

9. Have you had a previous diagnosis of cancer?

   ☐ Yes (Go to Q10)
   ☐ No

10. What type and when (nearest year)? ______________________

11. What type of genetic information was identified from lifepool?

- ☐ *ATM* gene change found
- ☐ *BRCA1* gene change found
- ☐ *BRCA2* gene change found
- ☐ *PALB2* gene change found
- ☐ *TP53* gene change found
- ☐ Genetic information that was relevant to my family and I
- ☐ A gene disposing to breast/ovarian cancer
- ☐ I can't recall/I'm not sure

What have you done since receiving genetic information from lifepool?

12. Have you attended an appointment at a Familial Cancer Centre or genetics service to have genetic counselling and testing?
   - ☐ Yes [Go to Q16]
   - ☐ No [Go to Q13]

13. Do you plan to attend a Familial Cancer Centre or genetics service to have genetic counselling and testing?
   - ☐ Yes [Go to Q14]
   - ☐ No [Go to Q15]
   - ☐ I haven’t decided

14. Please select from the following why you haven’t attended an appointment yet:
   - ☐ I have an appointment coming up
   - ☐ I have a referral but haven’t booked an appointment yet
   - ☐ I’m having trouble making an appointment
   - ☐ I plan to book an appointment but haven’t gotten around to it yet
   - ☐ Other_____________

15. Why aren’t you planning to attend an appointment?
   - ☐ I don’t have time
   - ☐ I’m not interested
   - ☐ I don’t need to
   - ☐ I don’t know how to organise an appointment
   - ☐ Other_____________

16. Have you had genetic testing for the gene change (mutation) identified by lifepool?
   - ☐ Yes
   - ☐ No [Go to Q17]

17. Why not?_________________________________

18. The following are ways to manage increased breast/ovarian cancer risk for women.
    Please indicate, from "I definitely won't do this" to "I already do/have done this", how you feel about the following statements:

	I'm not sure what this means	I definitely won't do this	I'm unsure if I will do this	I probably will do this	I definitely will do this	I already do/have done this
More frequent breast screening (e.g., annual breast MRI)						
Surgery to remove both breasts (bilateral risk-reducing mastectomy)						
Risk-reducing medication for breast cancer (e.g., tamoxifen or raloxifene)						
Surgery to remove both ovaries and fallopian tubes (bilateral risk-reducing salpingo-oophorectomy) [survey logic: *all options for Q11, except ATM or PALB2 mutations*]						

How do you feel about your risk of cancer?

19. How do you feel about your risk of cancer?

If you have previously developed cancer, please consider how you feel about your risk of getting cancer again.

Please indicate, from "disagree strongly" to "agree strongly", how you think about the following statements:

	Disagree strongly	Disagree mildly	Agree mildly	Agree strongly
1. I feel that I am going to get **breast** cancer	1	2	3	4
2. I feel that I am very vulnerable to **breast** cancer	1	2	3	4
3. I feel that I am going to get **ovarian** cancer [survey logic: *all options for Q11, except ATM or PALB2 mutations*]	1	2	3	4
4. I feel that I am very vulnerable to **ovarian** cancer [survey logic: *all options for Q11, except ATM or PALB2 mutations*]	1	2	3	4

20. What do you think your chance is of developing breast cancer in your lifetime?

    Please choose a number between 0% (no chance of breast cancer) and 100% (definitely will get breast cancer)

    Sliding scale here 0-100

21. What do you think your chance is of developing ovarian cancer in your lifetime?

    [survey logic: *all options for Q11, except ATM or PALB2 mutations*]

    Please choose a number between 0% (no chance of ovarian cancer) and 100% (definitely will get ovarian cancer)

    Sliding scale here 0-100

22. Please indicate, from *never* to *almost always*, how often you worry about cancer:

	Never	Sometimes	Often	Almost always
1. How often have you thought about your chances of getting cancer (again)?	1	2	3	4
2. Have these thoughts affected your mood?	1	2	3	4
3. Have these thoughts interfered with your ability to do daily activities?	1	2	3	4
4. How concerned are you about the possibility of getting cancer (again) one day?	1	2	3	4
5. How often do you worry about developing cancer (again)?	1	2	3	4
6. How much of a problem is this worry?	1	2	3	4
7. How often do you worry about the chance of family members developing cancer?	1	2	3	4
8. How concerned are you about the possibility that you will need surgery (again)?	1	2	3	4

[survey logic: *"again" appears for items 1, 4, 5 and 8 those who respond "yes" to Q9]*

How do you feel about your decision to participate in lifepool and receive genetic information?

23. The questions below are about some specific responses you may have had after receiving genetic information from lifepool.

    Please indicate from “never” to “often” , how often you have experienced each statement in the past week.

**Section 1**	Never	Rarely	Sometimes	Often
1. Feeling upset about my genetic information	0	1	3	5
2. Feeling sad about my genetic information	0	1	3	5
3. Feeling anxious or nervous about my genetic information	0	1	3	5
4. Feeling guilty about my genetic information	0	1	3	5
5. Feeling relieved about my genetic information	0	1	3	5
6. Feeling happy about my genetic information	0	1	3	5
7. Feeling a loss of control	0	1	3	5
8. Having problems enjoying my life because of my genetic information	0	1	3	5
9. Worrying about my risk of getting cancer again [survey logic: “again” appears if “yes” to Q9]	0	1	3	5
10. Being uncertain about what my genetic information means about my cancer risk	0	1	3	5
11. Being uncertain about what my genetic information means for my child(ren) and/or family’s cancer risk	0	1	3	5
12. Having difficulty making decision about cancer screening or prevention (e.g., having preventive surgery or having medical tests done)	0	1	3	5
13. Understanding clearly my choices for cancer prevention or early detection	0	1	3	5
14. Feeling frustrated that there are no definite cancer prevention guidelines for me	0	1	3	5
15. Thinking about my genetic information has impacted my work or family life	0	1	3	5
16. Feeling concerned about how my genetic information will affect my insurance status	0	1	3	5
17. Having difficulty talking about my genetic information with family members	0	1	3	5

18. Feeling that my family has been supportive during the process of learning my genetic information	0	1	3	5
19. Feeling satisfied with family communication about my genetic information	0	1	3	5
20. Worrying that my genetic information has brought about conflict within my family	0	1	3	5
21. Feeling regret about getting my genetic information	0	1	3	5

Section 2. *If you have children please answer Items 22 and 23. Otherwise, please go to Section 3.*

22. Worrying about the possibility of my children getting cancer	0	1	3	5
23. Feeling guilty about possibly passing on the gene to my child(ren)	0	1	3	5

Section 3. *If you currently have cancer, or have had it in the past, please answer Items 24 and 25. Otherwise, you are finished this section.*

24. Feeling that my genetic information has made it harder to cope with my cancer	0	1	3	5
25. Feeling that my genetic information has made it easier to cope with my cancer	0	1	3	5

How have you adapted to life after receiving genetic information from lifepool?

24. Please indicate, from "not at all" to "very much", how you think about the following statements.

Living with the genetic information provided by lifepool has…

	Not at all	A little bit	Somewhat	Quite a bit	Very much
1. Helped me accept the way things work out	1	2	3	4	5
2. Helped me learn to deal better with uncertainty	1	2	3	4	5
3. Taught me how to adjust to things I cannot change	1	2	3	4	5
4. Helped me to take things as they come	1	2	3	4	5
5. Helped me learn to handle difficult times	1	2	3	4	5
6. Helped me become more comfortable with who I am	1	2	3	4	5
7. Helped me become a stronger person	1	2	3	4	5
8. Helped me feel better about my ability to handle problems	1	2	3	4	5
9. Helped me become a better person	1	2	3	4	5
10. Helped relationships become more meaningful	1	2	3	4	5
11. Helped me become closer to the people I care about	1	2	3	4	5
12. Helped me become more aware of the love and support available from other people	1	2	3	4	5
13. Helped me learn my life is more meaningful	1	2	3	4	5
14. Given me a greater appreciation for life	1	2	3	4	5
15. Helped me develop a deeper sense of purpose in life	1	2	3	4	5

How have you talked about genetic information from lifepool with your family?

Some families share a lot with each other, some do not. We are interested in knowing if and how your family talks about the genetic information you have received from lifepool.

25. Have you told your family members about the genetic information from **life**pool?
    - ☐ Yes [Go to Q26]
    - ☐ No

26. Who have you told?

	None	Some	All
Sibling(s)	0	1	2
Parent(s)	0	1	2
Child(ren)	0	1	2
Cousin(s)	0	1	2
Grandparent(s)	0	1	2
Aunt(s) and uncle(s)	0	1	2
Niece(s) and nephew(s)	0	1	2

27. How have you talked about genetic information from **life**pool with family? Please indicate from "strongly agree" to "strongly disagree" how you feel about the following statements:

	Strongly agree	Agree	Neither	Disagree	Strongly disagree
I feel social pressure to tell my family members this genetic information	0	1	2	3	4
I would feel guilty if I did not tell my family members this genetic information	0	1	2	3	4
My family would not cope if I told them this genetic information	0	1	2	3	4
I can cope with telling my family this genetic information	0	1	2	3	4
I don't know enough about this genetic information to tell my family	0	1	2	3	4
I feel pressure from my family to tell them this genetic information	0	1	2	3	4
I would not tell my family members this genetic information because it might upset them	0	1	2	3	4
I am confident I could tell my family this genetic information	0	1	2	3	4
I feel pressure from health professionals to tell my family members this genetic information	0	1	2	3	4
I don't know how to tell my family members this genetic information	0	1	2	3	4

28. How have you talked about genetic information from **life**pool with family? Please indicate from "strongly agree" to "strongly disagree" what you think about the following statements:

	Strongly agree	Agree	Neither	Disagree	Strongly disagree
This genetic information is useful for my family members	0	1	2	3	4
My family and I are close	0	1	2	3	4
This genetic information is not relevant to my family members	0	1	2	3	4
My family and I don't talk about health/medical information	0	1	2	3	4
My family has a right to know this genetic information	0	1	2	3	4
It is my responsibility to share this genetic information with my family	0	1	2	3	4
This type of information is important to my family members	0	1	2	3	4
My family would understand this genetic information	0	1	2	3	4

[END SURVEY]

Thank you for taking the survey!

We sincerely appreciate you taking the time to participate in this study. Without you, projects like this would not be possible.

If you have any further questions, please do not hesitate to contact me on:

- Telephone: (03) 8559 6191
- Email: Laura.Forrest@petermac.org

Sincerely,

Dr Laura Forrest

Psychosocial Cancer Genetics Research Group

Parkville Familial Cancer Centre

Peter MacCallum Cancer Centre

Attachment E – Group 2 **life**pool psychosocial survey

[Note – this survey will be mounted onto REDCap, the formatting here is a guide only]

Information and consent to participate

You have been invited to participate in a study being conducted at the Peter MacCallum Cancer Centre that is investigating women's experiences of participating in **life**pool.

You have been invited to complete an **online questionnaire** because you attended Breast Screen Victoria and agreed to participate in the **life**pool study.

Lifepool is a research study investigating factors that contribute to breast cancer risk (including genes that predispose to women to develop breast and ovarian cancer). When you agreed to participate in **life**pool, you had a blood sample collected for the **life**pool research genetic studies.

We are inviting you to complete an **online questionnaire** because we are interested in your experience of participating in **life**pool and how you perceive your risk of cancer. Your opinions, views and experiences are valuable to us, and we greatly appreciate your participation.

Please read about the study in the information below. By clicking "Yes" at the bottom of this page you are providing consent to participate in this research study.

This study has been approved by the Peter Mac Human Research Ethics Committee.

What will I be asked to do?

Should you agree to participate, you will be asked to complete a **short online questionnaire**. The questionnaire has 2 sections and will take about 5-10 minutes to complete. You can access the questionnaire at the bottom of this page by clicking "Yes". You can complete the questionnaire over more than one session if needed. If so, you will need to make note of your individual Return Code to ensure your answers are saved and you continue where you left off.

There are no costs associated with participating in this research project, nor will you be paid. At the end of the questionnaire you can choose to download a PDF of your completed questionnaire.

How will my confidentiality be protected?

All identifying information about you will be removed so that it is de-identified. Your responses to the survey will be stored on a password-protected computer at the Peter MacCallum Cancer Centre in Melbourne, Australia for a minimum period of 5 years. At the end of this storage period all the data will be disposed of by deleting all computer files and backup files.

What are the benefits of participating?

By participating in this research study, you will be contributing to the support of individuals who receive genetic findings from research. There are no expected personal benefits to you the participant.

What are the risks of participating?

We do not expect there to be any risks from participating in this research and inform you of the outcome of the study via email.

Do I get a copy of the study findings?

At the end of the study we will publish relevant findings in peer-reviewed journals.

Where can I get further information?

Should you require any further information, or have any concerns about the content of this survey, please do not hesitate to contact:

Research Assistant:	Lyon Mascarenhas
Telephone:	(03) 8559 6189
Email:	Lyon.Mascarenhas@petermac.org
Available:	Monday, Tuesday and Friday (9am – 5pm)

Principal Investigator:	Dr Laura Forrest
Telephone:	(03) 8559 6191
Email:	Laura.Forrest@petermac.org
Available:	Tues, Wed & Thurs (9am – 5pm)

Should you have any concerns about the conduct of the project, you are welcome to contact the Executive Officer, Human Research Ethics, Peter MacCallum Cancer Centre (P: 03 8559 7540 | E: ethics@petermac.org).

How do I agree to participate?

By selecting the **YES** checkbox below, you agree to consent to participating in this study.

Consent to participate

Do you consent to participate?

☐ Yes

☐ No

About you

1. Have you had a previous diagnosis of cancer?

☐ Yes [END SURVEY]
☐ No

2. Please enter your date of birth (DD/MM/YYYY): _____________________

3. Please enter your postcode: _____________________

4. Please select your highest attained level of education:

☐ Year 11 or below

☐ Year 12

☐ Other Qualifications (non-school)

☐ Certificate level

☐ Advanced Diploma or Diploma

☐ Bachelor Degrees and above*

*includes Graduate Diplomas or Certificates and Postgraduate Degrees

5. What is your current household income?

☐ 0-$18,000

☐ $18,001 - $37,000

☐ $37,001 - $90,000

☐ $90,001 - $180,000

☐ $180,001 and over

6. What is your relationship status?

☐ Single

☐ Married or De facto

☐ Widowed

☐ Separated or Divorced

☐ Other:_____________

7. Do you have any children?

☐ Yes (go to Q8-9)

☐ No

8. How many daughters? ______________________

9. How many sons? ______________________

How do you feel about your risk of cancer?

10. How do you feel about your risk of cancer?

Please indicate, from "disagree strongly" to "agree strongly", how you think about the following statements:

	Disagree strongly	Disagree Mildly	Agree Mildly	Agree strongly
1. I feel that I am going to get **breast** cancer	1	2	3	4
2. I feel that I am very vulnerable to **breast** cancer	1	2	3	4
3. I feel that I am going to get **ovarian** cancer	1	2	3	4
4. I feel that I am very vulnerable to **ovarian** cancer	1	2	3	4

11. What do you think your chance is of developing breast cancer in your lifetime?
    Please choose a number between 0% (no chance of breast cancer) and 100% (definitely will get breast cancer)
    Sliding scale here 0-100

12. What do you think your chance is of developing ovarian cancer in your lifetime?
    Please choose a number between 0% (no chance of ovarian cancer) and 100% (definitely will get ovarian cancer)
    Sliding scale here 0-100

13. Please indicate, from *never* to *almost always*, how often you worry about cancer:

	Never	Sometimes	Often	Almost always
1. How often have you thought about your chances of getting cancer?	1	2	3	4
2. Have these thoughts affected your mood?	1	2	3	4
3. Have these thoughts interfered with your ability to do daily activities?	1	2	3	4
4. How concerned are you about the possibility of getting cancer one day?	1	2	3	4
5. How often do you worry about developing cancer?	1	2	3	4
6. How much of a problem is this worry?	1	2	3	4
7. How often do you worry about the chance of family members developing cancer?	1	2	3	4
8. How concerned are you about the possibility that you will need surgery?	1	2	3	4

[END SURVEY]

Thank you for taking the survey!

We sincerely appreciate you taking the time to participate in this study. Without you, projects like this would not be possible.

If you have any further questions, please do not hesitate to contact me on:

- Telephone: (03) 8559 6191
- Email: Laura.Forrest@petermac.org

Sincerely,

Dr Laura Forrest

Psychosocial Cancer Genetics Research Group

Parkville Familial Cancer Centre

Peter MacCallum Cancer Centre
